# *Helicobacter pylori* VacA toxin causes cell death by inducing accumulation of cytoplasmic connexin 43

**DOI:** 10.1038/cddis.2015.329

**Published:** 2015-11-12

**Authors:** K Yahiro, T Hirayama, J Moss, M Noda

**Affiliations:** 1 Department of Molecular Infectiology, Graduate School of Medicine, Chiba University, Chiba 260-8670, Japan; 2 Department of Bacteriology, Institute of Tropical Medicine, Nagasaki University, Nagasaki 852-8523, Japan; 3 Cardiovascular and Pulmonary Branch, NHLBI, National Institutes of Health, Bethesda, MD 20892, USA

The principles underlying pathogenicity of microbial toxins with pleiotropic effects have been studied by investigators with different areas of specialization; microbiology, immunology, physiology, pathology, cell biology and proteomics. Their diverse contributions and broad perspectives lead to the development of anti-toxin vaccines, such as those used for the prevention of diphtheria and tetanus and translated knowledge of basic mechanisms into therapeutic advances. Recent genomic, cell biological and molecular advances have enabled the determination of cellular targets and mechanism of action of bacterial toxins, including the elucidation of the molecular pathways by which toxins bind to cellular receptors, translocate and modify the functions of intracellular targets, leading to intoxication of the host cell. Bacterial toxins are classified into several families, for example, some toxins exert their effects at the cell surface by damaging host cell membranes (pore-forming toxins and super antigens).^1^ Other bacterial toxins such as diphtheria toxin cause cell death by ADP-ribosylation of a target protein, resulting in inhibition of protein synthesis.^1^ Toxins may alter target protein function by specific modifications, having a major impact on cell survival. Thereby, the pathological changes caused by bacterial toxins may be responsible for the disease caused by bacterial infection. A vaccine targeting the toxin may prevent the disease.

*Helicobacter pylori* (*H. pylori*) is a helical Gram-negative pathogen, which infects the human stomach in over 50% of the population of the world. Persistent infection causes gastric inflammation, ulcer and cancer.^2, 3^ *H. pylori* has multiple virulence factors that participate in the pathogenesis of the diseases. *H. pylori* produces an exotoxin, vacuolating cytotoxin (VacA), which is an important virulence factor associated with gastritis and ulceration. Indeed, oral administration of VacA to mice caused severe gastric damage.^3^ VacA consists of 33-kDa N-terminal domain involved in cytotoxicity and a 55-kDa C-terminal domain that binds to cell surface receptors. The primary sequence of VacA has no homology with any proteins.^4^ The secreted VacA assembles into a large flower-like hexameric or heptameric complex. The anion-channel activity of VacA is involved in multiple biological processes, resulting in vacuole formation, autophagy and mitochondrial damage, leading to apoptosis.^2, 3^ The detailed mechanisms by which VacA induces apoptosis and autophagy remain unknown.

A recent study showed that the expression level of connexin 43 (Cx43) in cells has an important role in VacA-induced cell death.^5^ Cx43, a member of the large human connexin (Cx) family, is ubiquitously expressed and a major component of gap junctions. It has a crucial role in intercellular communication, cell–cell channel formation and exchange of signaling molecules during development and in cell homeostasis.^6^ Our recent study explored the role of Cx43 in VacA-induced cell death and its presence in *H. pylori*-infected human gastric mucosa.^7^

It is known that Cxs in cultured cells undergo rapid turnover and have a short-life of about 1–5 h relative to other membrane proteins.^8^ Interestingly, human duodenum carcinoma cells incubated with VacA accumulated cytoplasmic Cx43, accompanied by LC3-II generation, caspase activation and poly(ADP-ribose)polymerase (PARP) cleavage, in a time- and dose-dependent manner. The levels of Cx43 mRNA were not altered by VacA, indicating that VacA disrupted Cx43 turnover without altering its synthesis. Consistent with a previous study,^5^VacA-induced apoptotic signals (e.g., caspase activation and PARP cleavage) were inhibited in Cx43-knockdown cells, which showed increased basal expression levels of apoptosis inhibitors, Bcl-2 and Bcl-xL. VacA-induced PARP cleavage was suppressed in FLAG-tagged Bcl-xL-overexpressing cells. Our findings suggest that VacA-induced cell death involves a unique pathway with increased cytoplasmic Cx43 accumulation.

Under normal conditions, Cx43 is localized at gap junctions in plasma membranes, whereas the increased Cx43 seen with VacA accumulated in cytoplasmic compartments and colocalized with several vesicle markers, for example, LC3, LAMP1, Atg16L1 and LysoTracker. These results indicate that Cx43 is associated with cellular trafficking pathways involving endosomes and autophagy. In Cx43-knockdown cells, VacA-induced LC3-II generation and formation of LysoTracker-positive vesicles were not inhibited, indicating that Cx43 was not involved in the pathway leading to VacA-induced autophagic vesicle formation. In contrast, knockdown of Atg16L1, which plays an essential role in autophagy,^9^ inhibited both Cx43 increase and LC3-II generation in VacA-treated cells as compared with control cells, suggesting that Atg16L1 is not only involved in LC3-II generation but also in Cx43 accumulation by VacA. Thus, VacA-increased Cx43 accumulated in a cytoplasmic fraction via effects on an autophagy signaling pathway. We further found that VacA-increased cytoplasmic Cx43 was colocalized with VacA in vesicles characterized by cholesterol-rich, detergent-resistant membranes. By localization of Cx43 with VacA in detergent-resistant membranes, degradation of Cx43 through an endosome/autophagy pathway might be suppressed, followed by an increase in cytoplasmic Cx43, leading to apoptotic cell death.

We explored if the reactive oxygen species/Rac1/ERK signaling pathway regulates both VacA-increased Cx43 and LC3-II generation. Prior study showed that VacA suppressed the turnover rate of intracellular GSH by impairing GSH metabolism.^10^ N-acetyl-cysteine, an antioxidant and free radical scavenger, significantly suppressed VacA-induced Cx43 increase and LC3-II generation. VacA-induced ERK phosphorylation and Rac1 activation were suppressed in N-acetyl-cysteine-treated cells. Inhibition of ERK and Rac1 activities suppressed VacA-induced Cx43 accumulation and LC3-II generation. In agreement, knockdown of ERK and Rac1 by siRNAs reduced Cx43 increase and LC3-II generation by VacA. VacA-induced ERK phosphorylation was suppressed by inhibition or knockdown of Rac1. Interestingly, ERK knockdown significantly suppressed VacA-induced PARP cleavage. These data indicated that GSH level controls Rac1/ERK activation, which in turn regulate VacA-increased Cx43 and LC3-II generation.

As described above, channel activity of VacA is critical for its biological activity. The chloride channel inhibitor, DIDS, significantly suppressed VacA-induced ERK phosphorylation, Cx43 increase and LC3-II generation. These results indicate that VacA-mediated channel activity is a key trigger to initiate these events.

Finally, we investigated Cx43 content in human gastric biopsies. Our data showed that Cx43 expression was barely detectable in gastric mucosa of *H. pylori*-negative patients. In contrast, Cx43 was elevated in the gastric epithelium of *H. pylori*-positive biopsy specimens. Our study provides new insights into the role of Cx43 in *H. pylori* infection as shown in Figure 1. Cx43 is a potential clinically relevant target in gastric inflammation and ulceration.

## Figures and Tables

**Figure 1:**
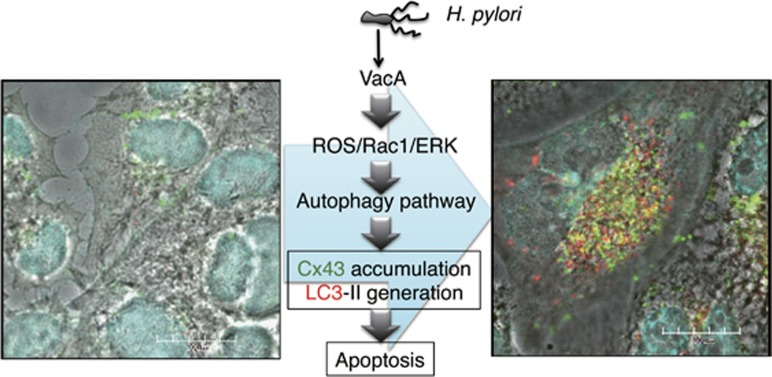
VacA induces Cx43 increase, autophagy and apoptosis. VacA binds and is internalized by cells. Toxin channel activity impairs GSH metabolism (reactive oxygen species), activation of Rac1, and ERK phosphorylation. These signal transduction events lead to enhanced Cx43 endocytosis. Cx43 accumulated in cytoplasmic compartments through effects of toxin on pre-autophagy pathways and colocalized with autophagosomal marker LC3. Cells were incubated with 120 nM heat-inactivated (left panel) or wild-type VacA (right panel) for 10 h and then reacted with anti-Cx43 (green) and anti-LC3 antibodies (red) and were stained with DAPI (cyan). Bars represent 20 *μ*m. The parts of yellow indicate the colocalization of Cx43 and LC3
